# *Toxoplasma gondii* ROP5 Enhances Type I IFN Responses by Promoting Ubiquitination of STING

**DOI:** 10.3390/ijms252011262

**Published:** 2024-10-19

**Authors:** Qi-Wang Jin, Ting Yu, Ming Pan, Yi-Min Fan, Ceng-Ceng Ge, Xiao-Bing He, Jing-Zhi Gong, Jian-Ping Tao, Bao-Quan Fu, Zhi-Zhong Jing, Si-Yang Huang

**Affiliations:** 1 Jiangsu Key Laboratory of Zoonosis, Jiangsu Co-Innovation Center for Prevention and Control of Important Animal Infectious Diseases and Zoonosis, Institute of Comparative Medicine, College of Veterinary Medicine, Yangzhou University, Yangzhou 225009, China; qiwangj@163.com (Q.-W.J.); yutingyt2023@163.com (T.Y.);; 2 State Key Laboratory for Animal Disease Control and Prevention, Lanzhou Veterinary Research Institute, Chinese Academy of Agricultural Sciences, College of Veterinary Medicine, Lanzhou University, Lanzhou 730000, China

**Keywords:** *Toxoplasma gondii*, ROP5, cGAS-STING pathway, ubiquitination

## Abstract

*Toxoplasma gondii* is a widely spread opportunistic pathogen that can infect nearly all warm-blooded vertebrates and cause serious toxoplasmosis in immunosuppressed animals and patients. However, the relationship between the host’s innate immune system and effector proteins is poorly understood, particularly with regard to how effectors antagonize cGAS-STING signaling during *T. gondii* infection. In this study, the ROP5 from the PRU strain of *T. gondii* was found to promote cGAS-STING-mediated immune responses. Mechanistically, ROP5 interacted with STING through predicted domain 2 and modulated cGAS-STING signaling in a predicted domain 3-dependent manner. Additionally, ROP5 strengthened cGAS-STING signaling by enhancing the K63-linked ubiquitination of STING. Consistently, ROP5 deficient PRU (PRUΔROP5) induced fewer type I IFN-related immune responses and replicated faster than the parental strain in RAW264.7 cells. Taken together, this study provides new insights into the mechanism by which ROP5 regulates *T. gondii* infection and provides new clues for strategies to prevent and control toxoplasmosis.

## 1. Introduction

*Toxoplasma gondii* is a widely distributed intracellular protozoan parasite capable of infecting nearly all warm-blooded vertebrates, including humans [1,2,3]. *T. gondii* primary infection in children and adults is asymptomatic in most patients. The most typical clinical manifestation is isolated cervical or occipital lymphadenopathy during chronic infection. Very infrequently, myocarditis, polymyositis, pneumonitis, hepatitis, or encephalitis can arise in immunocompromised individuals during acute toxoplasma infection [3]. The lifecycle of *T. gondii* is unique, and acute and chronic phases could characterize the progression of the infection. During the acute phase, the replication of the dominant tachyzoite stage leads to parasite dissemination. As immune responses develop, this rapid proliferation is controlled and converts into slow-growing bradyzoites, which settle in long-lasting tissue cysts in the brain and muscles. In immunocompetent adults, the infection is typically persistent but asymptomatic. However, in immunocompromised individuals, *T. gondii* infection can lead to life-threatening toxoplasmosis [3,4].

It has long been appreciated that innate immune responses, in initial phases, are critical for the outcomes of *T. gondii* infections [5,6,7,8,9]. A key principle that underlies resistance to infection is the ability of pattern recognition receptors (PRRs) to sense pathogens and initiate innate immune responses. Previous research has shown that many toll-like receptors (TLRs) play critical roles in recognizing pathogen-associated molecular patterns (PAMPs) during *T. gondii* infection [10,11,12,13]. Nod-like receptors (NLRs) have been equally well-characterized [14,15,16]. In addition to these canonical PRRs, an increasing number of studies have shown that the cytoplasmic sensor cGMP-cAMP synthase (cGAS) plays an essential role during *T. gondii* infection. Wang et al. reported that cGAS- or stimulator of interferon genes (STING)-deficient mice are markedly more susceptible to *T. gondii* than wild-type (WT) mice [17]. Chen et al. recently reported that the PRU strain could inhibit cGAS-STING signaling in RAW264.7 cells [18]. Additionally, in our previous study, we found that the replication of *T. gondii* is significantly enhanced in cGAS-STING signaling-deficient RAW264.7 cells. However, the precise mechanisms by which cGAS-STING signaling defends against *T. gondii* infection are not fully understood.

The cGAS-STING signaling axis detects pathogenic DNA to trigger an innate immune response, primarily involving a strong type I interferon (IFN) reaction against microbial infections [19]. cGAS can recognize and be activated by DNA ligands, which include canonically single-stranded and double-stranded microbial DNA, as well as self-mitochondrial DNA [19]. Once activated, cGAS synthesizes cyclic GMP-AMP (cGAMP), which transmits a signal to the STING [20,21]. STING, an adaptor molecule localized in the endoplasmic reticulum (ER), recruits TANK-binding kinase 1 (TBK1) upon activation. TBK1 phosphorylates IRF3/7, which in turn initiates the transcription of type I IFNs [22,23,24]. It is also believed that the recruited TBK1 leads to the expression of pro-inflammatory cytokines via NF-κB activation [25,26,27]. The released type I IFNs and pro-inflammatory cytokines can recruit inflammatory cells and enhance adaptive immune responses to defend against *T. gondii* infection.

The secreted effectors of *T. gondii*, primarily originating from the parasite’s rhoptries (named ROPs) and dense granules (named GRAs), have been defined to be injected into host cells, where they play a crucial role in modulating host immune responses [17,18,28,29]. Few *T. gondii* effectors have been characterized as participating in the modulation of cGAS-STING signaling. The GRA15 protein from ME49 strains enhances host defense responses by promoting STING polyubiquitination and oligomerization [17], while ROP18 from the PRU strain inhibits host innate immunity by interacting with IRF3 [18]. In a previous study, we showed that ROP16 from the PRU strain inhibits the cGAS-STING pathway by suppressing the polyubiquitination of STING. During the *T. gondii* invasion process, various effectors are secreted into the host cytosol. The role of other effectors in modulating the cGAS-STING pathway is yet to be uncovered. The ROP5 protein belongs to the pseudo-kinase family, which lacks enzymatic activity but acts as a central scaffold for ROP17 and ROP18 to phosphorylate several IRGs, primarily originating *T. gondii* virulence [30,31,32,33]. ROP5 is secreted during invasion and can be exposed to host cell cytoplasm [34,35]. Notably, each parasite strain carries a unique number and type of ROP5 genes, diversifying ROP5 coordination as a whole [36,37]. Type I and Ⅲ strains share a similar complement of ROP5 alleles necessary for acute virulence. In contrast, type Ⅱ strains contain a distinct cluster of ROP5 alleles associated with lower virulence [37,38]. ROP5 from type I and type Ⅲ strains has been shown to promote the replication of *T. gondii* through the ROP5-ROP18-IRGs pathway, while the ROP5 from type Ⅱ strains failed to promote the activation of ROP5-ROP18-IRGs axis and led to lower virulence in mice [30,33,38]. Therefore, ROP5 from type Ⅱ strains may play an important role through other signaling pathways, possibly leading to type Ⅱ *T. gondii* being the most epidemic strain worldwide.

The cGAS-STING axis plays a key role in sensing PAMPs during *T. gondii* infection. Many effectors secreted by *T. gondii* play important roles in modulating innate immune responses. In our previous screening experiment [28], we primarily found that ROP5 from the PRU could enhance cGAS-mediated immune responses, although the mechanism remained unclear. The present study aims to characterize the function of ROP5 in regulating *T. gondii* infection through the cGAS-STING pathway and explore the mechanism by which ROP5 modulates this signaling. To this end, we generated the ROP5-deficient PRU and identified the molecular targets of ROP5. In this study, we identified that ROP5 enhances type-I IFN responses by potentiating STING activity during PRU infection, which provides new insights into the mechanism by which ROP5 regulates *T. gondii* infection.

## 2. Results

### 2.1. T. gondii ROP5 Enhances cGA-Mediated Immune Responses

**Figure 1:**
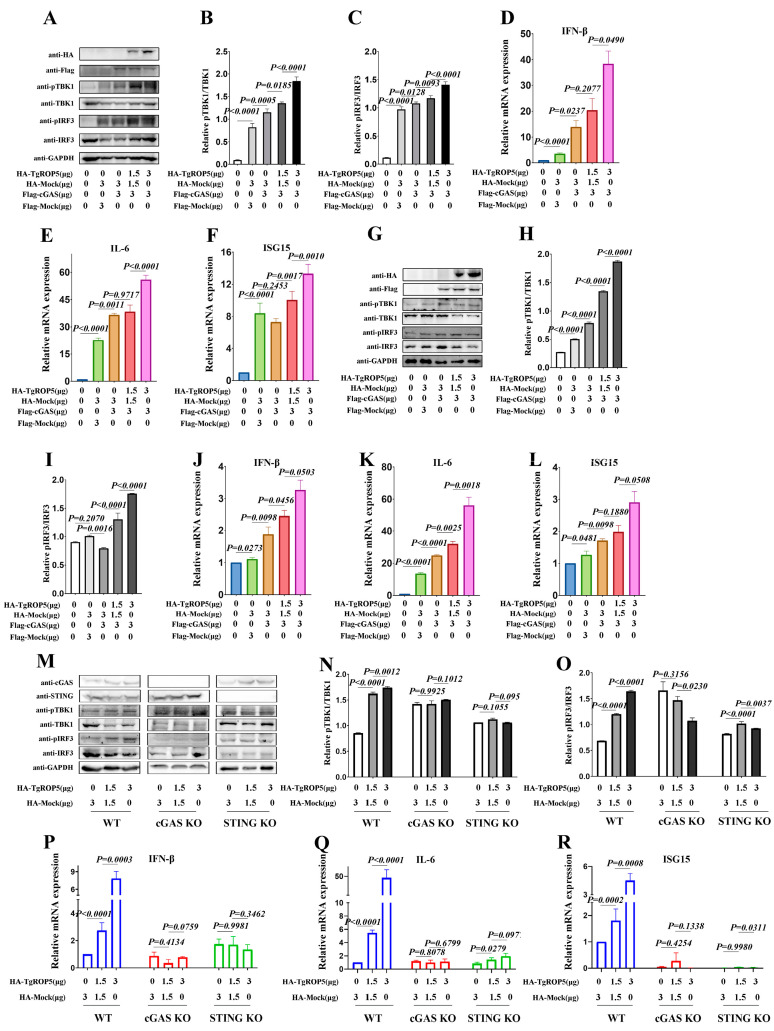
ROP5 enhances the activation of cGAS-STING signaling. (**A**–**F**) RAW264.7 cells were co-transfected with the indicated cocktail of plasmids; 48 h past transfection, the cells were collected for Western blotting assays; 24 h past transfection, the cells were harvested for qRT-PCR assays. The cGAS-STING signaling was detected by Western blotting for the indicated proteins (**A**). Image J densitometry analysis for pTBK1 relative to total TBK1 and pIRF3 relative to total IRF3 according to the Western blotting results (**B**,**C**). Relative mRNA levels of IFN-β, IL-6 and ISG15 were evaluated with qPCR methods (**D**–**F**). (**G**–**L**) BMDM cells were co-transfected with the indicated cocktail of plasmids; 48 h past transfection, the cells were collected for Western blotting assays; 24 h past transfection, the cells were harvested for qRT-PCR assays. (**M**–**R**) RAW-Lucia ISG (WT), RAW-KO-cGAS (cGAS KO), and RAW-KO-STING (STING KO) cells were co-transfected with indicated cocktail of plasmids; 48 h past transfection, the cells were collected for Western blotting assays; 24 h past transfection, the cells were harvested for qRT-PCR assays. The primary antibodies used for the Western blotting assays were in 1:1000 dilution, while the secondary antibodies used for the Western blotting assays were in 1:10,000 dilution. The data represent the results of three independent experiments with three biological replicates or three independent experiments with similar results. Bars and error bars show as mean ± SD of three independent experiments. Statistical analysis was performed with Student’s *t*-test.

Initially, we examined the modulation of the cGAS-STING signaling pathway by ROP5 using Western blotting. The results showed that in RAW264.7 cells co-transfected with cGAS- and TgROP5-encoding plasmids, phosphorylated TBK1 (pTBK1) and phosphorylated IRF3 (pIRF3) were significantly enhanced in a dose-dependent manner compared to the control cells (Figure 1A–C). Furthermore, we measured the mRNA expression of pro-inflammatory cytokines induced by the cGAS-STING signaling pathway using qRT-PCR. The results revealed that the expression of IFN-β, IL-6, and ISG15 was significantly upregulated by ROP5 in a dose-dependent manner in RAW264.7 cells (Figure 1D–F). These results indicate that ectopically expressed ROP5 can promote cGAS-STING-mediated immune responses in RAW264.7 cells. Additionally, we assessed cGAS-STING signaling and the expression of cytokines modulated by ROP5 in BMDMs. The differentiation rate of BMDMs was determined using the FCA method, and the results showed BMDM purity of over 96% (Figure S2). Similarly, pTBK1 and pIRF3 were significantly enhanced in a dose-dependent manner compared to control cells (Figure 1G–J). The mRNA expression of IFN-β, IL-6, and ISG15 was also significantly upregulated by ROP5 in a dose-dependent manner in BMDMs (Figure 1J–L). These results indicate that ectopically expressed ROP5 promotes cGAS-STING-mediated immune responses in macrophages. Next, we evaluated ROP5’s modulation of immune responses in cGAS-STING pathway-deficient cells. The cGAS-STING signaling pathway was analyzed via Western blotting in WT and cGAS-STING pathway-deficient RAW264.7 cells. As expected, the results showed that, compared with the lower-dose and higher-dose ROP5-transfected groups, ROP5 promoted pTBK1 and pIRF3 in WT RAW264.7 cells but failed to do so in cGAS- or STING-deficient cells (Figure 1M–O). The mRNA expression of IFN-β, IL-6, and ISG15 was measured via qRT-PCR. As confirmed, the mRNA expression of IFN-β, IL-6, and ISG15 was significantly upregulated by ROP5 in WT RAW264.7 cells, whereas these enhancements were completely absent in cGAS- or STING-deficient cells compared with single cGAS-transfected cells with ROP5- and cGAS-co-transfected cells (Figure 1P–R). These results indicate that ectopically expressed ROP5 enhances immune responses in a cGAS-STING-dependent manner. Taken together, ROP5 promotes cGAS-STING signaling, thereby enhancing immune responses.

### 2.2. ROP5 Is Required for Mounting cGAS-STING-Mediated Responses During PRU Infection

**Figure 2:**
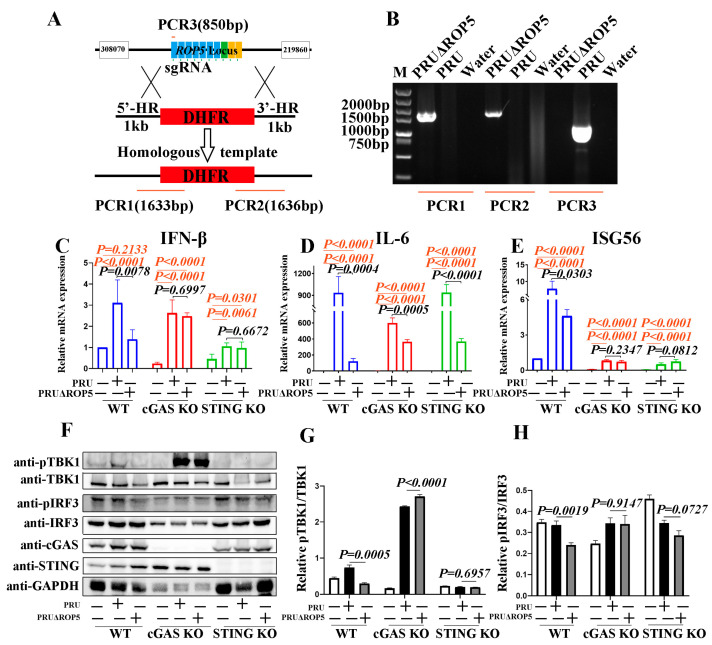
ROP5 deficiency inhibits cGAS-STING signaling-mediated immune responses. (**A**) The schematic diagram of CRISPR/Cas9-mediated DHFR insertion at the ROP5 locus and diagnostic PCRs on ROP5 knockout PRU tachyzoites. The ROP5 locus is located between the TGME49_308070 (chrXII: 554,350...562,936 bp) and TGME49_219860 (chrXII: 578,740...590,285 bp) genes. We constructed the knockout plasmid for homologous integration into the parasite line PRU by targeting downstream of TGME49_308070 (chrXII: 554,350...562,936 bp) and upstream of TGME49_219860 (chrXII: 578,740...590,285 bp), which could ensure that all the ROP5 alleles could be deleted. (**B**) PCR products of diagnostic PCRs used to identify the PRU mutants. (**C**–**H**) WT, cGAS KO, and STING KO RAW264.7 cells were infected with indicated PRU or PRUΔROP5 tachyzoites; 8 h past infection, the cells were harvested for qPCR assays; 12 h past infection, the cells were collected for Western blotting assays. Relative mRNA levels of IFN-β (**C**), IL-6 (**D**), and ISG56 (**E**) in infected cells were evaluated with qPCR methods. The cGAS-STING signaling was detected by Western blotting for the indicated proteins (**F**). Image J shows the densitometry analysis for pTBK1 relative to total TBK1 and pIRF3 relative to total IRF3 according to the Western blotting results (**G**,**H**). The primary antibodies used for the Western blotting assays were in a 1:1000 dilution. While the secondary antibodies used for western blotting assays were in 1:10,000 dilution. The data represent the results of three independent experiments with three biological replicates or three independent experiments with similar results. Bars and error bars show as mean ± SD of three independent experiments. Statistical analysis was performed with Student’s *t*-test. The variable “*p*” in black font means the significance between PRU- and PRUΔROP5-infected cells, while the variable “*p*” in orange means the significance between infected and non-infected cells.

Previous reports have identified the important role that the cGAS-STING pathway plays during *T. gondii* infection, and our previous findings demonstrated that ectopically expressed ROP5 could promote cGAS-STING signaling in RAW264.7 cells. However, the functions of endogenous ROP5 in modulating cGAS-STING signaling remained unclear. We next focused on the function of endogenous ROP5 during *T. gondii* infection. Initially, we constructed ROP5 knockout (KO; PRUΔROP5) tachyzoites using the CRISPR-Cas9 method, as illustrated in Figure 2A. We measured the KO efficiency through PCR, with the schematic shown in Figure 2A. The results indicated that ROP5 was completely knocked out in PRUΔROP5 tachyzoites (Figure 2B). Subsequently, we assessed the immunomodulatory functions of endogenous ROP5 in WT or cGAS-STING pathway-deficient RAW264.7 cells by infecting the cells with ROP5-deficient *T. gondii*. The qPCR results showed a reduction in the mRNA expression levels of IFN-β and ISG56 in the WT RAW264.7 cells infected with PRUΔROP5 compared to those infected with PRU or PRUΔROP5 (Figure 2C–E, left panel). In contrast, in cGAS-STING pathway-deficient cells, the impaired immune response induced by PRUΔROP5 infection was restored (Figure 2C,E, middle and right panels). Notably, PRUΔROP5 infection led to lower IL-6 expression in all three cell types compared with PRU infection (Figure 2D). These results indicate that endogenous ROP5 promotes the expression of IFN-β and ISG56 in a cGAS-STING pathway-dependent manner. Finally, we examined the function of endogenous ROP5 in modulating cGAS-STING signaling through Western blot analysis. Consistently, the activation of cGAS-STING signaling, as indicated by the pTBK1/TBK1 and pIRF3/IRF3, was significantly reduced in PRUΔROP5-infected WT cells (Figure 2F–H, left panel). In contrast, the activation of TBK1 and IRF3 were not decreased in PRUΔROP5-infected cGAS KO or STING KO cells (Figure 2F–H, middle and right panel). These results indicate that endogenous ROP5 enhances cGAS-STING signaling. Altogether, *T. gondii* infection induces type I IFN-related immune responses, which can be modulated by ROP5 through the cGAS-STING pathway. 

### 2.3. T. gondii ROP5 Interacts with STING 

**Figure 3:**
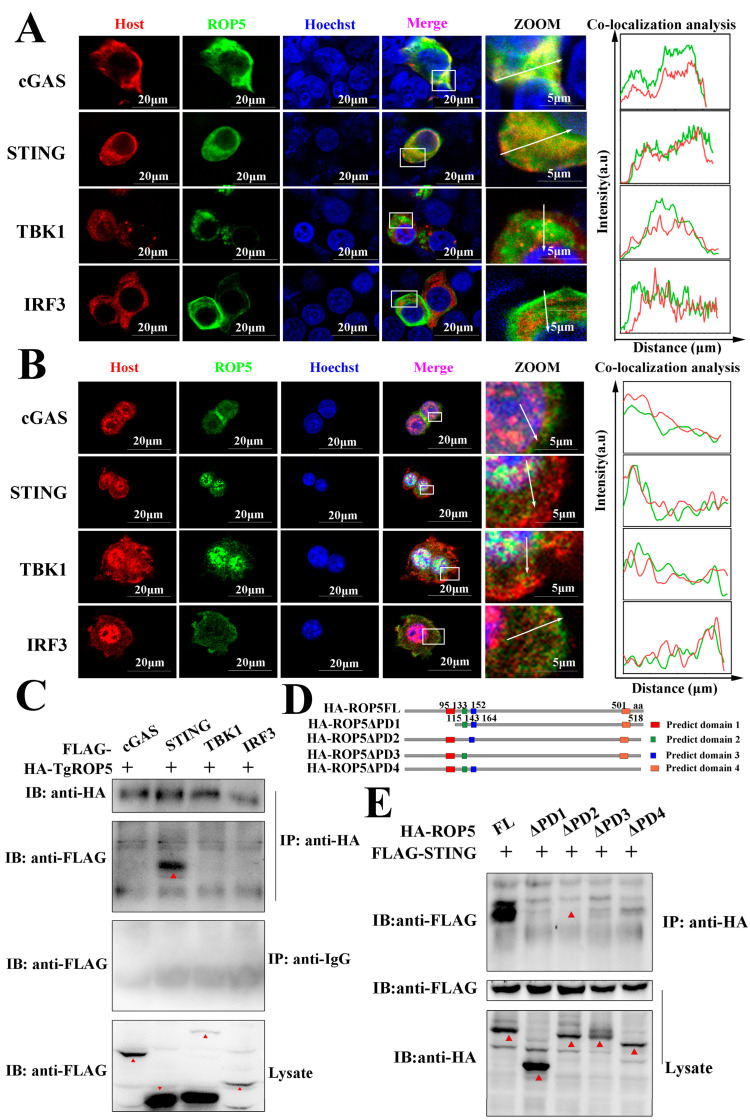
*T. gondii* ROP5 interacts with STING. (**A**) Subcellular locations of ROP5 were explored with IFA in HEK293T cells. HEK293T cells were cultured on coverslips and co-transfected with HA-ROP5 and indicated host proteins (FLAG-cGAS, FLAG-STING, FLAG-TBK1, FLAG-IRF3) encoding plasmids using Lip2000 reagents. At 24 hpt, the cells were measured by immunofluorescence. HA-ROP5 proteins were stained with Alexa Flour^TM^ 488 goat anti-mouse IgG (green), while the indicated host proteins were stained with Alexa Flour^TM^ 594 goat anti-rabbit IgG (red) and the nuclei were stained with Hoechst (blue). (**B**) Subcellular locations of ROP5 were explored with IFA in RAW264.7 cells. RAW264.7 cells were cultured on coverslips and co-transfected with HA-ROP5 and indicated host proteins (FLAG-cGAS, FLAG-STING, FLAG-TBK1, FLAG-IRF3) encoding plasmids using the Gene Pulser Xcell Electroporation System (Bio-Rad Hercules, CA, USA). At 24 hpt, the cells were measured by immunofluorescence. HA-ROP5 proteins were stained with Alexa Flour^TM^ 488 goat anti-mouse IgG, while the indicated host proteins were stained with Alexa Flour^TM^ 594 goat anti-rabbit IgG and the nuclei were stained with Hoechst. (**C**) Screening for ROP5 interaction host proteins by Co-IP method. RAW264.7 cells were co-transfected with ROP5 and indicated host protein encoding plasmids using the Gene Pulser Xcell Electroporation System. At 24 hpt, the cell lysates were collected and analyzed by Co-IP and Western blotting methods with the indicated antibodies. (**D**) Schematic representation of the mature form of *T. gondii* ROP5 and truncated constructs used in this study. (**E**) Screening for the crucial domain of ROP5 when interacting with STING. RAW264.7 cells were co-transfected with indicated ROP5 and truncations and host STING encoding plasmids using the Gene Pulser Xcell Electroporation System. At 24 hpt, the cell lysates were collected and analyzed using Co-IP and Western blotting methods with the indicated antibodies. For Western blotting assays, the primary antibodies used were in 1:1000 dilution and the secondary antibodies used for Western blotting assays were in 1:10,000 dilution. For IFA, the primary antibodies used were in 1:1000 dilution and the secondary antibodies used for Western blotting assays were in 1:1000 dilution. For Co-IP, the primary antibodies used were in 1:200 dilution. The targeted blotting stripes were indicated with red triangles. The co-localization analysis was performed by ImageJ V1.8.0 (National Institutes of Health, Bethesda, MD, USA). The green and red solid lines in the graph represent the intensity of green or red fluorescence. The data represent the results of three independent experiments with similar results.

To elucidate the potential mechanism by which TgROP5 modulates cGAS-STING signaling, we sought to identify TgROP5’s molecular targets. We performed immunofluorescence to analyze the subcellular localization of ROP5 in HEK293T and RAW264.7 cells by sequentially co-transfecting plasmids encoding ROP5 and host proteins. The results showed that ectopically expressed ROP5 proteins were diffusely distributed in the cytoplasm of both HEK293T and RAW264.7 cells, where ROP5 co-localized with cGAS, STING, TBK1, and IRF3 in HEK293T cells (Figure 3A) and RAW264.7 cells (Figure 3B). To further identify potential target(s), we analyzed RAW264.7 cells co-transfected with ROP5 and host proteins encoding plasmids using the co-immunoprecipitation (co-IP) method. As shown in Figure 3C, TgROP5 specifically interacted with host STING proteins but not with cGAS, TBK1, or IRF3 in RAW264.7 cells.

To investigate which domain is integral for the TgROP5-STING interaction, we analyzed ROP5 using online tools http://smart.embl-heidelberg.de/, accessed on 3 August 2021, and four truncated mutants of ROP5, including HA-ROP5ΔPD1 (Predict domain 1), HA-ROP5ΔPD2, HA-ROP5ΔPD3, and HA-ROP5ΔPD4, were constructed (Figure 3D and Figure S3). We co-transfected Flag-STING with the truncated ROP5 individually into the RAW264.7 cells and used the co-IP method for detection. The results shown that compared with the full-length, the ROP5ΔPD1, ROP5ΔPD3, and ROP5ΔPD4 truncations displayed weaker interactions with the STING proteins. In contrast, the ROP5ΔPD2 truncation failed to interact with STING (Figure 3E). These results suggested that the PD1, PD3, and PD4 play regulatory roles in mediating the ROP5-STING interaction, while PD2 is the crucial domain to mediate the interaction between the ROP5 and STING. Taken together, we demonstrated that ROP5 interacted with STING, which is mediated by the PD2 of ROP5.

### 2.4. T. gondii ROP5 Enhances cGAS-STING-Mediated Immune Responses Dependent on PD3

**Figure 4:**
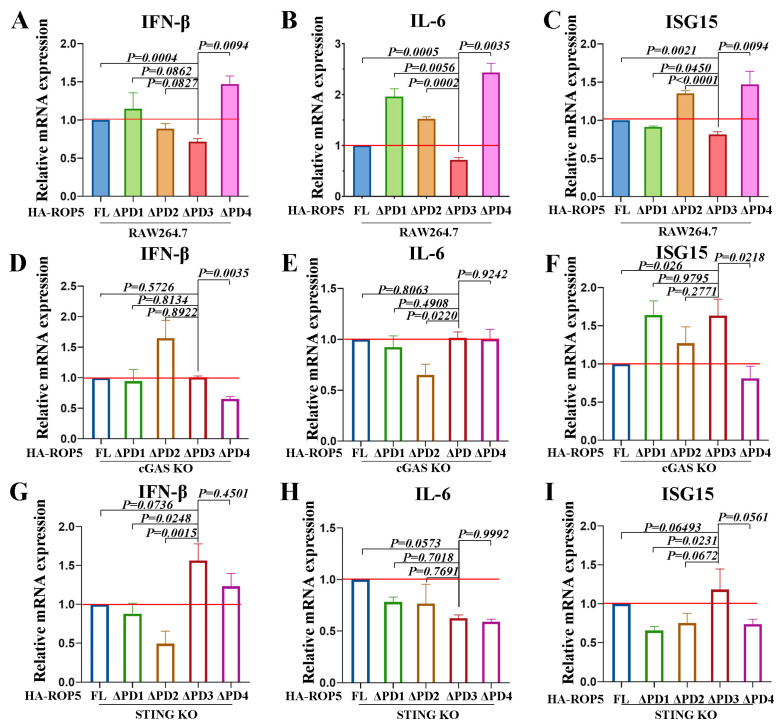
*T. gondii* ROP5 modulation of cGAS-STING signaling depends on the predict domain 3. WT, STING KO and cGAS KO RAW264.7 cells were transfected with full-length or indicated truncations encoding plasmids using the Gene Pulser Xcell Electroporation System; 24 h past transfection, the cells were harvested for qRT-PCR assays. (**A**–**C**) Relative mRNA levels of IFN-β, IL-6, and ISG15 in WT cells were evaluated with qPCR methods. (**D**–**F**) Relative mRNA levels of IFN-β, IL-6, and ISG15 in cGAS KO cells were evaluated with qPCR methods. (**G**–**I**) Relative mRNA levels of IFN-β, IL-6, and ISG15 in STING KO cells were evaluated with qPCR methods. The data represent the results of three independent experiments with three biological replicates. Bars and error bars show as mean ± SEM of three independent experiments. Statistical analysis was performed with Student’s *t*-test. The baseline of relative value 1 was labeled in red.

To further clarify the molecular mechanism by which ROP5 modulates cGAS-STING signaling, we elucidated the functional domain responsible for enhancing cGAS-STING pathway-related immune responses. Full length ROP5 and four truncated mutants of ROP5 were transfected into WT, cGAS KO, and STING KO RAW264.7 cells, respectively, and the associated cytokine levels were evaluated through qRT-PCR. The results showed that, compared with other proteins, the ROP5ΔPD3 mutant induced lower mRNA expression of IFN-β, IL-6, and ISG15 in RAW264.7 cells (Figure 4A–C). However, in cGAS-deficient cells, the impaired type I IFN-related immune responses induced by the ROP5ΔPD3 mutant were restored (Figure 4D–F). Similarly, the ROP5ΔPD3 mutant induced comparable type I IFN-related immune responses to the full-length protein and other mutants in STING-deficient cells (Figure 4G–I). These results indicate that PD3 of ROP5 is critical for enhancing cGAS-STING-mediated immune responses. 

### 2.5. T. gondii ROP5 Promotes K63-Linked Ubiquitination and STING Activation 

**Figure 5:**
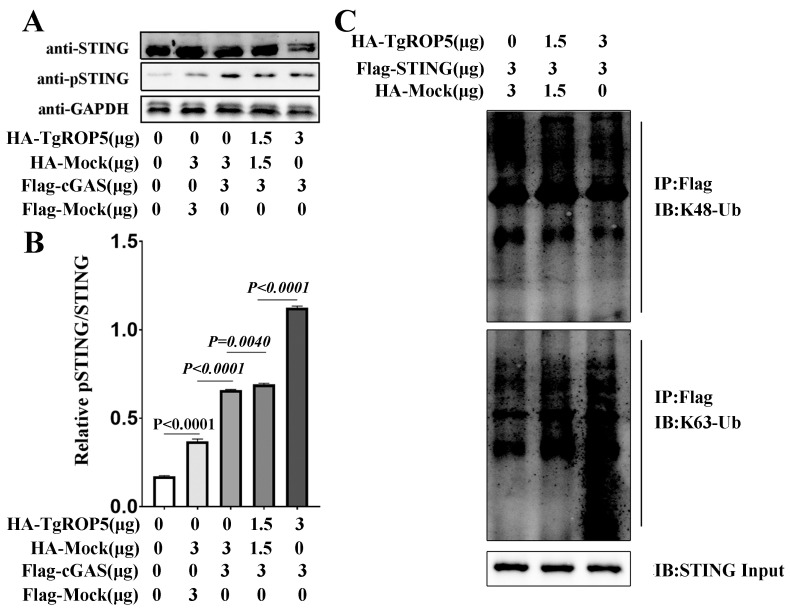
*T. gondii* ROP5 enhancing the K63-linked polyubiquitination of STING. RAW264.7 cells were co-transfected with the indicated cocktail of plasmids using the Gene Pulser Xcell Electroporation System; 48 h past transfection, the cells were harvested for Western blotting and IP assays. (**A**) Cell lysates were detected by Western blotting with indicated antibodies. (**B**) Image J shows the densitometry analysis for pSTING relative to total STING. (**C**) The equal amount of lysate was primarily immunoprecipitated with anti-Flag antibodies and finally detected by Western blotting with anti-K48 and anti-K63linkage-specific Polyubiquitin antibodies. For Western blotting assays, the primary antibodies used were in 1:1000 dilution and the secondary antibodies used for Western blotting assays were in 1:10,000 dilution. For Co-IP, the primary antibodies used were in 1:200 dilution. The data represent the results of three independent experiments with similar results. Bars and error bars show as mean ± SEM of three independent experiments. Statistical analysis was performed with Student’s *t*-test.

Previous reports have shown that pTBK1 can phosphorylate STING at its CTT domain, providing a docking site for IRF3. In turn, IRF3 is phosphorylated, dimerizes, and gains transcriptional activity. Therefore, the phosphorylation of STING is a hallmark of its activation, leading to the induction of type I IFN responses. To evaluate the activation of STING, we co-transfected RAW264.7 cells with cGAS and ROP5-encoding plasmids and detected pSTING and STING through Western blotting. As shown in Figure 5A,B, ROP5 promoted the phosphorylation of STING in a dose-dependent manner. These results demonstrate that ROP5 enhances cGAS-STING signaling by modulating the activation of STING rather than increasing its abundance. Accumulating evidence suggests that post-translational modifications (PTMs) of STING modulate its activity and protein stability. Polyubiquitination is a key PTM of STING, with K48-linked polyubiquitin chains primarily targeting proteins for proteasomal degradation, while K63-linked polyubiquitin regulates protein functions. To determine whether ROP5 affects the polyubiquitination of STING, we co-transfected RAW264.7 cells with HA-ROP5 and Flag-STING encoding plasmids and purified Flag-STING using the immunoprecipitation method. Polyubiquitination of STING was evaluated by Western blotting with polyubiquitin-specific monoclonal antibodies, including those specific to K48-linked and K63-linked polyubiquitin, respectively. The results showed that ROP5 dose dependently increased the K63-linked polyubiquitination of STING (Figure 5C). In contrast, ROP5 did not strongly induce K48-linked polyubiquitination of STING. Therefore, ROP5 promotes K63-linked ubiquitination of STING, modulating its activation.

### 2.6. ROP5 Plays Crucial Roles in Anti-T. gondii Immune Responses 

**Figure 6:**
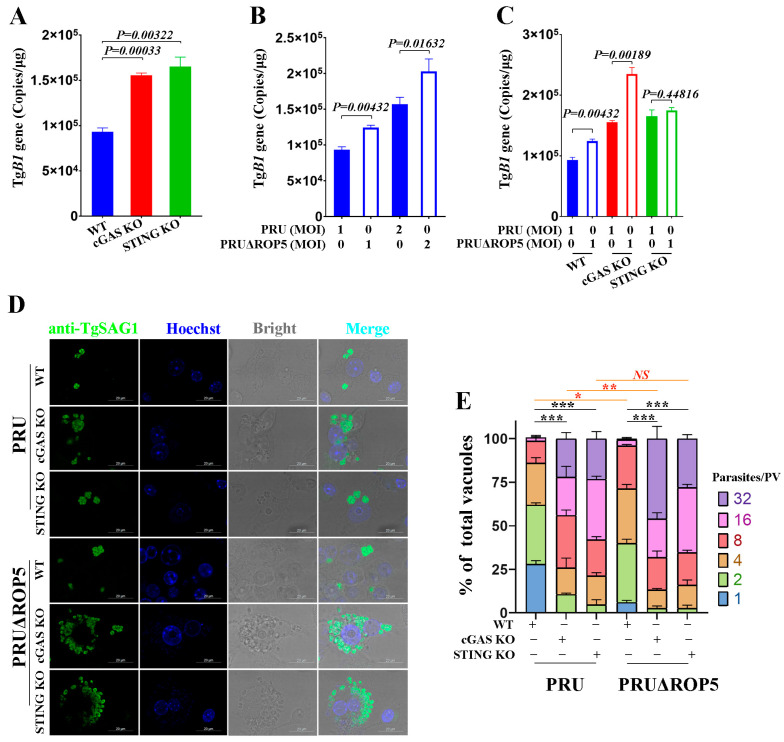
*T. gondii* ROP5 plays an important role in defense against PRU infection WT, STING KO, and cGAS KO RAW264.7 cells were infected with PRU or PRUΔROP5 tachyzoites at an indicated MOI; 48 h past infection the cells were harvested and assayed by qPCR for copies of TgB1 gene. (**A**) qPCR detected the TgB1 gene in PRU-infected RAW264.7, RAW-KO-cGAS, and RAW-KO-STING cells. (**B**) qPCR detected the TgB1gene in RAW264.7 cells infected by PRU or PRUΔROP5 tachyzoites with different MOI. (**C**) qPCR detected the TgB1 gene in WT, STING KO, and cGAS KO RAW264.7 cells infected by PRU or PRUΔROP5 tachyzoites. (**D**) The WT, STING KO, and cGAS KO RAW264.7 cells were cultured on coverslips and infected with PRU or PRUΔROP5 tachyzoites, respectively. At 48 hpi, the replication of tachyzoites in different cells was detected by IFA. The *T. gondii* SAG1 proteins were stained with Alexa Flour^TM^ 488 goat anti-mouse IgG (1:1000), while the nuclei were stained with Hoechst (1:1000). (**E**) The number of parasites in each parasitophorous vacuole (PV) was valued based on the IFA results. The primary antibodies used for IFA were in 1:1000 dilution, while the secondary antibodies used for Western blotting assays were in 1:1000 dilution. The data represent the results of three independent experiments with three biological replicates or three independent experiments with similar results. Bars and error bars show as mean ± SEM of three independent experiments. Statistical analysis was performed with Student’s *t*-test. * 0.01 < *p* < 0.05; ** *p* < 0.01; *** *p* < 0.001, and *NS p* ≥ 0.05.

Since ROP5 can modulate cGAS-STING signaling to promote type-I IFN responses, we further explored its functions in PRU infection. We primarily investigated the role of cGAS-STING signaling in PRU replication and the results were consistent with our previous findings. As shown in Figure 6A, PRU load was significantly increased in cGAS-STING pathway-deficient cells, including RAW-KO-cGAS and RAW-KO-STING cells, compared to the RAW264.7 cells group. These results suggested that cGAS-STING signaling is critical for defense against PRU infection. To further investigate the functions of ROP5 in PRU infection, we infected RAW264.7 cells with PRU and PRUΔROP5 strains, respectively. The results showed that the PRUΔROP5 caused a significantly higher load in different MOIs (Figure 6B). These results demonstrate that ROP5 plays an important role in modulating PRU infection in RAW264.7 cells. Furthermore, we evaluated whether ROP5 controls the infection of PRU through cGAS-STING signaling by infecting cGAS-STING pathway-deficient cells with PRUΔROP5. We found that the PRUΔROP5-induced higher PRU load was lost in RAW-KO-STING cells, while in RAW-KO-cGAS cells, the function of ROP5 in modulating PRU infection was retained (Figure 6C). These results suggested that in line with the function of ROP5 in modulating type I IFNs responses, ROP5 might regulate the PRU infection through cGAS-STING signaling in a STING-dependent manner. To further confirm the role of ROP5 in regulating PRU replication through cGAS-STING signaling, we performed immunofluorescence analysis in PRU-infected RAW264.7 cells. Corresponding to the PRU load, which was detected with qPCR methods, the results showed that the PRU replicated more rapidly in cGAS-STING pathway-deficient cells. Furthermore, PRUΔROP5 grew more rapidly in RAW264.7 and RAW-KO-STING cells compared with PRU-infected groups. Moreover, while in RAW-KO-STING cells, the ROP5-deficient PRU showed a replication capacity similar to its normal one (Figure 6D,E). Taken together, these results demonstrated that ROP5 plays a key role in regulating PRU replication in macrophages by modulating cGAS-STING-mediated immune responses.

## 3. Discussion

Innate immune responses provide a powerful first line of defense against the infection of pathogens, including *T. gondii*. It is well established that the responses of type Ⅱ IFNs (IFN-γ), mediated by STAT1, play a major role in killing or limiting the replication of *T. gondii*. Recently, the effects of type I IFNs (IFN-β/α) on controlling *T. gondii* infection have also been confirmed [17,18,39]. In this study, we focused on the functions of cGAS-STING pathway-induced type I IFNs in defending against *T. gondii* infections and confirmed that the cGAS-STING signaling is critical to limit PRU replication. cGAS-STING signaling provides powerful defense responses against *T. gondii* infection. Previous studies have shown that PRU has evolved multiple strategies to modulate this signaling to allow parasite growth and persistence, while avoiding excessive parasite burden, which can kill the host [40]. In the present study, we found that the effector of ROP5 from the PRU strain functioned as a positive regulator of type I IFNs. On the one hand, the enhanced-type I IFNs induced by ROP5 could control this infection well to avoid severe symptoms. On the other hand, strong-type I IFNs responses could limit the quick replication of PRU at acute phases and progress to chronic phases to establish lifelong infections. Taken together, these effects of ROP5 benefit both *T. gondii* and the host.

The ROP5 protein belongs to the pseudo-kinase family and is encoded by a polymorphic gene with 6–11 tandem duplicates. It is the only significant virulence effector identified between type I and type Ⅱ strains of *T. gondii* in mice [36,37,38,41]. Deletion of ROP5 in the type I strain significantly attenuates virulence in mice but does not affect the replications in vitro. The level of IFN-γ may be the main reason for inducing these contradictory effects of ROP5 in different infection models, because it is well identified that ROP5I controls virulence by regulating the active kinase of ROP18 to phosphorylate the IFN-γ induced GTPases [31,32,33]. Sequencing analysis indicates that type I and type III strains contain similar ROP5 alleles, while the ROP5 alleles in the type II strain differ significantly. Previous studies indicated that ROP5 I and III efficiently promote the degradation of IRGs mediated by ROP18, whereas ROP5 II fails to enhance the function of ROP18 in vitro and in vivo [38,41]. The functions and mechanisms of ROP5 in type I and type III strains are well understood, but the role of ROP5 in type II strains remains unknown. In this study, we found that deletion of ROP5 accelerated the replication of PRU in RAW264.7 cells. Moreover, we demonstrated a novel mechanism by which ROP5 II modulates PRU infection in RAW264.7 cells.

ROP5 is encoded in a multicopy locus, arranged as a tandem array of 11 copies in type Ⅱ strains. Previous studies have revealed that the ROP5 locus is not a single functional unit; individual isoforms can significantly alter virulence in the absence of other copies [33,36,37,38]. In our study, to disrupt the entire ROP5 locus, we used sequences from the type II PRU strain, corresponding to downstream of TGME49_308070 (chrXII: 554,350...562,936 bp) and upstream of TGME49_219860 (chrXII: 578,740...590,285 bp), to construct a KO plasmid for homologous integration into the PRU parasite line (Figure 2A shows a schematic of the approach used to create the PRUΔROP5 parasite), ensuring that all ROP5 alleles could be deleted. ROP5 paralogs are highly divergent. Previous sequencing analysis indicated that the ROP5 locus of the type II strain contains four unique isoforms. However, the ROP5 isoforms of the type II strain are quite conserved. For example, there are only eight nonsynonymous polymorphisms, all located just N-terminal to the site of frame between the ROP5B (minor) and ROP5C (major) [37,38]. In our research, we amplified the ROP5 type II coding sequences (CDS) using PCR, with primers designed based on TGME49_308090 (https://toxodb.org/ (accessed on 20 August 2021)). We were unable to differentiate between the ROP5B and ROP5C alleles in the PRU strain, and thus, we refer to them collectively as ROP5 type II in this study.

Cytokines are soluble mediators that facilitate cell-to-cell communication in immune responses. In this study, two cytokines were analyzed. IFN-β is induced by the MyD88 and STING is mediated signaling pathways, while IL-6 is primarily induced by MyD88 signaling [42,43,44]. IFN-β is a typical type-I IFN, which limits *T. gondii* replication through interferon-stimulated gene (ISG) products in infected macrophages and DCs, including ISG15, which was detected in this research. IL-6 is a prototypical cytokine with characteristic redundant and pleiotropic activities, which defend against *T. gondii* infection by inducing the differentiation of B and T cells in infected animals [45,46,47,48]. In this study, we found that ROP5 modulates the expression of IL-6 (Figure 1E,K,Q and Figure 2D); however, this modulation occurs in a cGAS-STING signaling-independent manner (Figure 1Q and Figure 2D). It is well established that NF-κB and STAT1 modulate the mRNA expression of IL-6 [42,44,49]. STAT1 signaling is predominantly initiated by IFNs, while the activation of NF-κB is the hallmark of TLR signaling [50]. We speculated that ROP5 regulates IL-6 by targeting different signaling pathways. In cGAS-STING pathway intact cells, ROP5 promoted IFN-β to induce STAT1-mediated IL-6 expression (Figure 1E,K). While in cGAS-STING signaling-deficient cells, ROP5 enhances TLR signaling to induce NF-κB-mediated IL-6 expression (Figure 1Q and Figure 2D). 

*T. gondii* is an obligate intracellular protozoan parasite that can inject a large amount of PAMPs into host cells during infection. A few PAMPs have been identified as being recognized by different PRRs. Apart from cGAS-STING pathway, many PRRs have been identified as taking part in sensing *T. gondii* during infection. The complex composition of *T. gondii* PAMPs might contribute to the role of ROP5 in modulating *T. gondii* replication via a STING-dependent but cGAS-independent mechanism (Figure 6C–E). STING is a dual-functional molecule; in addition to its role as an adaptor in cGAS-initiated signaling, STING can also function as a receptor and be directly activated by cGAMP. This study suggests cGAMP as a component of *T. gondii* PAMPs, which may directly initiate STING signaling independently of cGAS. In summary, ROP5 regulates cGAS-STING signaling by targeting STING, and the absence of STING completely disrupts the modulatory function of ROP5. 

The ROP5 family is a closely related group of polymorphic pseudo-kinases that lack conserved canonical kinase domains, yet they have been identified as important regulators of signaling networks. They may function as molecular scaffolds for effectors and their downstream targets, or by modulating the activity of catalytically active enzymes [32,36]. In this study, we predicted the domain of ROP5 based on the predicted protein sequence of ROP5 (TGME49_308090) using an online tool (http://smart.embl-heidelberg.de/ (accessed on 3 August 2021)). No canonical domains were detected; however, four low-complexity regions were identified in the diagram. These features of ROP5 were consistent with a previous report, which demonstrated that ROP5 influences *T. gondii* virulence through a conserved non-canonical motif. We constructed various truncated mutants based on these predicted features to explore the interaction and functional motif/domains of ROP5. We found that PD2 was crucial for mediating the interaction with STING (Figure 3D,E), while PD3 was essential for modulating cGAS-STING signaling, which mediates immune responses (Figure 4A–I). Notably, a PD2-deficient ROP5, despite failing to bind STING, retained the ability to modulate immune responses. This suggests that the distinct functional and interactional domains of ROP5 might arise from an indirect interaction between ROP5 and STING. Consistent with previous studies, as a pseudo-kinase, ROP5 regulates signaling by recruiting effectors and active enzymes [30,31,33]. In our study, ROP5 likely regulated STING activation by recruiting DUBs.

Recent studies have increasingly shown that the polyubiquitination of STING modulates its activity and protein stability. The UL13 protein of PRV promotes K27-linked ubiquitination of STING to degrade STING by recruiting RNF5 [51]. Host SEL1L–HRD1 promoted the K27-linked ubiquitination of STING to control STING mediated innate immunity [52]. SARS-CoV2 PLpro deubiquitinated K63-linked ubiquitinated STING at Lys289 to suppress type I interferon responses [53]. The GRA15 from ME49 strains of *T. gondii* promoted STING polyubiquitination at Lys337 to enhance host defense immune responses [17]. Our previous study indicated that ROP16 from the PRU strain of *T. gondii* inhibited the K63-linked ubiquitination of STING to regulate type I IFN-related immune responses. In the present study, we found that ROP5 from the PRU strain promoted K63-linked polyubiquitination of STING to enhance host defense responses (Figure 5). Furthermore, the ROP5 is a pseudo-kinase without ubiquitination-related functions. Therefore, we speculate that ROP5 modulates the polyubiquitination of STING by recruiting related enzymes. The exact molecular mechanism will be elucidated in future studies.

In conclusion, we identified *T. gondii* ROP5 as inhibiting PRU replication by modulating cGAS-STING-mediated immune responses by enhancing the K63-linked polyubiquitination of STING. Although the role of *Toxoplasma gondii* ROP5 in regulating the cGAS-STING pathway has been clarified in this study, the specific mechanism has not yet been clarified. As a protein kinase, ROP5 contained no ubiquitinase or de-ubiquitinase activity. Therefore, which effector enzymes does ROP5 recruit to regulate the ubiquitination of STING? How does ROP16 regulate effector enzymes and how do effector enzymes regulate STING ubiquitination? We attempt to answer these questions in our further study. Our findings provide new insights into the novel function of ROP5 and its role in *T. gondii* infection, which provide a new theoretical approach for designing vaccines to prevent and control toxoplasmosis.

## 4. Materials and Methods

### 4.1. Cells and Parasite Culture

The RAW264.7, HEK293T, and HFF cells were originally obtained from ATCC and maintained in our laboratory. The RAW-Lucia ISG (mouse macrophages), RAW-Lucia ISGKO-cGas (cGAS knockout IRF-inducible Lucia luciferase reporter mouse macrophages), and RAW-Lucia ISG-KO-STING (STING knockout IRF-inducible Lucia luciferase reporter mouse macrophages) were originally purchased from InvivoGen Company (San Diego, CA, USA). The BMDMs (mouse bone marrow-derived monocytes) were isolated and cultured according to the protocol reported by Davis et al. [54], which was conducted with the ethics approval of the Ethics Committee of the Yangzhou University (approval no. 202302071).

The parasite used in this research was PRU, an extensively used type Ⅱ strain of *T. gondii* with much less pathogenicity, and was maintained in our laboratory. The PRU strains of *T. gondii* could stimulate stronger proinflammatory responses in mice, which help to curb parasite dissemination and parasite-induced pathology [38].

The cells were cultured in Dulbecco’s Modified Eagle Medium (DMEM, GIBCO) and supplemented with 10% FCS (GIBCO), 2 mM L-glutamine (GIBCO) and 100 U/mL penicillin, and 100 μg/mL streptomycin (GIBCO) at 37 °C under 5% atmospheric CO_2_.

The PRU strain of *T. gondii* were generated in HFF cells according to our previous study [28].

BMDM were generated from C57BL/6J mice according to a protocol described by Beckley K. Davis [39] and with the ethics approval of the Ethics Committee of the Yangzhou University (approval no. 202302071).Mice were anesthetized with isoflurane and euthanized by cervical dislocation. Femurs and tibias were dissected and placed in phosphate buffered saline (PBS) on ice. The ends of the bones were removed and flushed with PBS to remove the whole marrow. Cells were mechanically dissociated using a pipette and washed by centrifugation. Marrow was then resuspended in macrophage differentiation media (DMEM supplemented with 20% L929 conditioned media, 10% heat-inactivated FBS, 1% L-glutamine, 1% sodium pyruvate, 1% nonessential amino acids, and 1% penicillin/streptomycin). It was incubated for 7 days in a 5% CO 2 humidified tissue culture incubator. We checked the cells daily and added fresh media (5 mL) every 2–3 days. The BMDM were harvested for flow cytometry analysis and further experiments.

### 4.2. Antibodies, Reagents, and Plasmids

The primary antibodies used for western blotting, including Rabbit-cGAS (#31659), Rabbit-STING (#13647), Rabbit-pSTING (#72971), Rabbit-TBK1 (#3504), Rabbit-pTBK1 (#5483), Rabbit-IRF3 (#4302) and Rabbit-pIRF3 (#4947), Rabbit-anti-K63-linkage-specific polyubiquitin (#5621), and Rabbit-anti-K48-linkage-specific polyubiquitin (#8081) were purchased from Cell Signaling Technology (Danvers, MA, USA). Mouse-GAPDH (#G8795), Mouse-FLAG (#F3165), HRP-Goat-Rabbit IgG (#A0545), HRP-Goat-mouse IgG (#A4416) antibodies, and Hoechst 33258 (#94403) were obtained from Sigma-Aldrich (St. Louis, MO, USA). Rabbit-HA (#ab137838), Mouse-HA (#ab49969), and Rabbit-FLAG (#ab205606) antibodies were obtained from Abcam (Cambridge, UK). Mouse-*T. gondii* SAG1 (#MA5-18268), Alexa Flour^TM^ 594-goat-rabbit IgG (#A11012), Alexa Flour^TM^ 488 -goat-rabbit IgG (#A11008), and Alexa Flour^TM^ 488-goat-mouse IgG (#A11001) were obtained from Invitrogen (Carlsbad, CA, USA). FITC anti-mouse/human CD11b Antibody (#101205), and PE anti-mouse F4/80 Antibody (#123110) were obtained from Biolegend (San Diego, CA, USA).

Protein A/G Plus-Agarose (#sc-2003) was purchased from SANTA CRUZ Biotechnology (Dallas, TX, USA). TIANamp Genomic DNA Kit (#DP304) was purchased from TIANGEN (Beijing, China). HiScript^®^ III RT SuperMix for qPCR Kits (#R323) and AceQ Universal SYBR qPCR Master Mix Kits (#Q511) were purchased from Vazyme (Nanjing, China). RIPA Lysis Buffer (#P0013), which mainly contained 50 mM Tris (pH 7.4), 150 mM NaCl, 1% Triton X-100, 1% sodium deoxycholate, 0.1% SDS, and a variety of inhibitors such as sodium orthovanadate, sodium fluoride, and EDTA was purchased from Beyotime (Shanghai, China). The ECL reagent (#P2300) was purchased from NCM Biotech (Suzhou, China).

The Flag-tagged proteins encoding plasmids, which involved in cGAS-STING pathway, including Flag-cGAS, Flag-STING, Flag-TBK1, and Flag-IRF3, were constructed in our previous study [28]. The full-length and truncated ROP5 were amplified by PCR using PRU cDNA as template and then were cloned into the pCDNA3.1-3× HA vectors. All plasmids were verified by DNA sequencing. The primers used for PCR were listed in Table S1.

### 4.3. Transfection and Infection of Cells

RAW264.7 cell transfection, including normal and genetically modified cells, was performed using the Gene Pulser Xcell Electroporation System (Bio-Rad), following the protocols from the manufacturer. The HEK-293T cell transfections were performed with Lipofectamine 2000 reagents (Invitrogen) and according to the protocols from the manufacturer. 

The *T. gondii* infection experiments were operated according our pervious report [28]. In brief, the PRU tachyzoites were harvested and washed three times with PBS to remove the component of host cells. The purified tachyzoites were resuspended and added into appropriate cells (~1 × 106/1 mL/well in total) at a dose of MOI = 1 (1 × 106/well) or 2 (2 × 106/well) according to the report of Chen Min et al. [18]. The uninvaded PRU were removed by washing with PBS at 2 hpi and the infected cells were cultured in appropriate conditions for further evaluation with different methods.

### 4.4. Generation of ROP5-Deficient PRU (PRUΔROP5)

The ROP5 gene in the PRU strain was knocked out using the CRISPR-Cas9 system according to the protocol reported by Shen Bang et al. [55,56] and Behnke MS et al. [33]. In brief, the ROP5-encoding gene TGME49_308090 that targeted single-guide RNA (sgRNA, GATCGGTCACCGACTCGAAG) was amplified and modified into pSAG1::CAS9-U6:sgUPRT plasmids (these were gifted by Prof. Shen Bang of Huazhong Agricultural University, and were originally available from Add gene) using a Q5 site-directed mutagenesis kit (New England Biolabs, Ipswich, MA, USA) with primers listed in Table S1. The homologous template was obtained using the ClonExpress II one-step cloning kit (Vazyme Biotech Co., Ltd., Nanjing, China) to connect the homologous arms at the 3′ and 5′ ends of ROP5 gene amplified using genomic DNA of PRU as a template. The DHFR+ resistance cassette was amplified using pUPRT::DHFR-D plasmids (these were gifted by Prof. Shen Bang of Huazhong Agricultural University, and were originally available from Add gene) as a template for pUC19. The resulting plasmid was identified by nucleotide sequence analysis, and the 5HR-DHFR-3HR homologous fragment was amplified using the positive plasmid as a template. Furthermore, 20 μg of ROP5-specific CRISPR-Cas9 plasmids and 100 μg of the purified homologous fragment of 5HR-DHFR-3HR were mixed and co-transfected into freshly egressed tachyzoites of PRU using the Gene Pulser Xcell Electroporation System (Bio-Rad) and then obtained in HFF cells. Subsequently, the PRU were selected 3 times with 3 μM pyrimethamine (Sigma-Aldrich, USA) and individual parasite clones were obtained by culturing in a 96-well tissue culture plate using a limiting dilution method. Finally, the clonal mutants were identified with PCR methods and the positive clonal of PRUΔROP5 were collected to infect appropriate cells for further research.

### 4.5. Quantitative Real-Time Reverse Transcription PCR (qRT-PCR)

For relative quantification assays, 1 μg of total RNA, isolated from infected or transfected cells with TRIzol regents, was reverse transcribed into cDNA with HiScript^®^ III RT SuperMix for qPCR Kits. The cDNA was quantified using AceQ Universal SYBR qPCR Master Mix Kits. The CT values of different housekeeping genes including GAPDH, TuBa, Actb, and TBP were measured and analyzed. According to the CTs and the expression stability in different cells, the GAPDH gene was defined to work as an internal control (Figure S1). The relative fold changes of mRNAs were calculated with the comparative threshold cycle (2^−ΔΔCt^) method according to the previous report [57]. For absolute quantification assay, the pMD19-T-TgB1 plasmids (constructed in our previous study [28]) were used as the standard substance and diluted into different folds for standard curve plotting, as described previously [28]. The total gDNA was isolated from *T. gondii*-infected cells with a TIANamp Genomic DNA Kit. One microgram genomic DNA was used for qRT-PCR and the copies of TgB1 were calculated using the plotted standard curve. The primers used for qPCR are listed in Table S1.

### 4.6. Immunofluorescence Assay

The transfected or infected cells (HEK293T and RAW264.7 cells) were cultured on coverslips for a further immunofluorescence assay, as described previously [28]. Firstly, the cells were fixed with 4% paraformaldehyde and followed by permeabilized with 0.1% Triton X-100. Then, the non-specific epitopes were blocked with 10% FBS diluted into PBS for 1 h and the primary antibodies (diluted according to the protocols from the manufacturer) were incubated at 4 °C overnight. Finally, the fluorescence-conjugated secondary antibody (for 1:1000 dilution) and Hoechst (for 1:1000 dilution) were incubated for 1 h in darkness. Confocal fluorescence microscopy was performed using a Leica SP8 FALCON microscope (Leica Microsystems, Wetzlar, Germany) equipped with a Leica TCS SP8 X scanner.

### 4.7. Co-Immunoprecipitation (Co-IP) Assay

The plasmid-transfected RAW264.7 cells were harvested and lysed with 0.5 mL RIPA Lysis Buffer for 30 min on ice. The cell lysates were further treated with ultrasonication and clarified by centrifugation. For each immunoprecipitation, the cell lysates (0.5 mL) were incubated with the rabbit antibodies (1 μG) at 4 °C for 1 h on a 3D shaker. The lysates were further incubated overnight with Protein A/G PLUS-Agarose (15 μL) at 4 °C on a 3D shaker. The protein-bund beads were then collected and washed using a RIPA Buffer (0.75 mL) at least 6 times. The final washed beads were resuspended in 50 μL of 1× sample buffer (GenScript, Nanjing, China) and boiled for 10 min for further evaluation.

### 4.8. Western Blotting Assay

The cellular lysis or Co-IP samples were loaded to 12% SDS-polyacrylamide gels for electrophoresis and transferred onto a polyvinylidene fluoride (PVDF) membrane (EMD Millipore, Darmstadt, Germany). The membrane was blocked with 5% skimmed milk in TBS at 4 °C overnight and then incubated with the appropriate primary antibodies at 4 °C for 8–10 h. After being washed 4 times with TBST, the membrane was incubated with HRP-conjugated secondary antibodies at room temperature for 1 h and washed 6 times with TBST. All membranes were visualized with the ECL reaction buffer and imaged with a Tanon automatic chemiluminescence image analysis system according to the manufacturer protocols (Tanon, China).

### 4.9. Statistical Analysis

All quantitative data were demonstrated as the mean and standard deviation (SD), and analyzed using GraphPad Prism8 (GraphPad software Inc., Boston, MA, USA). Statistical significance between two groups was analyzed by unpaired Student’s *t*-test, one-way analysis of variance (ANOVA), and using GraphPad Prism8. At least three biological replicates were induced.

## Data Availability

The data that support the findings of this study are available in the methods and/or Supplementary Material of this article.

## Supplementary Materials

The following supporting information can be downloaded at: https://www.mdpi.com/article/10.3390/ijms252011262/s1.
