# Supplementary material for: Toxoplasma gondii ROP5 Enhances Type I IFN Responses by Promoting Ubiquitination of STING
Source: Int J Mol Sci. 2024 Oct 19;25(20):11262. doi: 10.3390/ijms252011262 (PMC11508707; doi:10.3390/ijms252011262)
Supplement: Supplementary file 1 [file ijms-25-11262-s001.zip › ijms-3186142-supplementary.pdf]

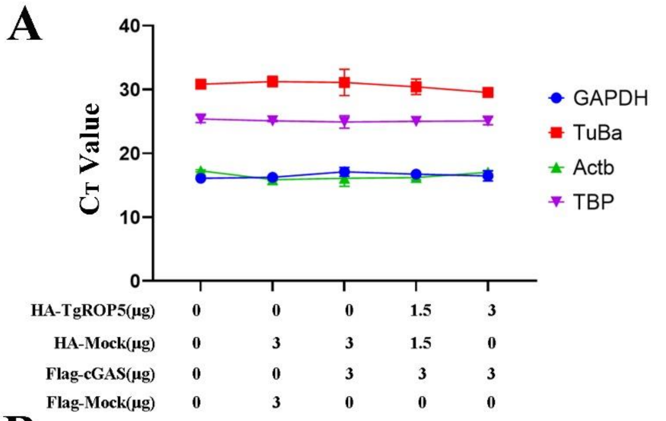

B

Target markers	GAPDH	TuBa	Actb	TBP
Minimum	16.10	29.53	15.87	24.91
Maximum	17.10	31.24	17.27	25.37
Range	1.002	1.713	1.394	0.4604
Mean	16.52	30.62	16.49	25.08
Std Deviation	0.4001	0.6884	0.6139	0.1720
Std Error of Mean	0.1789	0.3079	0.2745	0.07694

Figure S1 | Different housekeeping genes expressed in different cells 1 μg of total RNA, isolated from different cells with TRIzol regents, was reverse transcribed into cDNA with HiScript® III RT SuperMix for qPCR Kits. The cDNA was quantified using AceQ Universal SYBR qPCR Master Mix Kits. The CT values of different housekeeping genes including GAPDH, TuBa, Actb and TBP were measured and analyzed. (A) The CT value in different cells. (B) Descriptive statistics of different genes expressing.

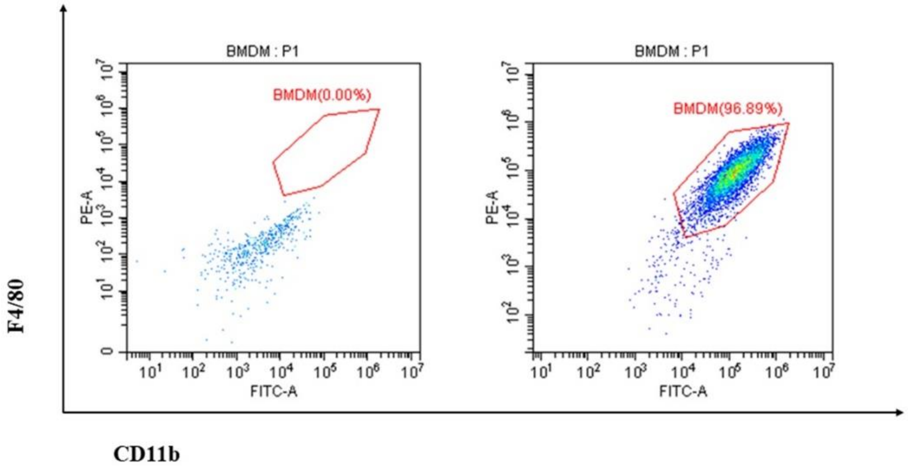

Figure S2 | Flow CytoMetry analysis of BMDM BMDM were generated from C57BL/6J mice and resuspended in macrophage differentiation media for 7 days in a 5% CO 2 humidified tissue culture incubator. The BMDM were harvested for FCA. The Flow CytoMetry analysis was performed using a BD AccuriTM C6 Plus flow cytometer (BD Biosciences). All data sets were analyzed with Flowjo software (TreeStar, Ashland, OR, United States).

A

>ROP5 FL

MATKLARLATWLVLVGCLLWRAGAVQLSPPNSRTNDLASGTPHVARGDTEAQSGTGDDSDFPQGVVEEV
ADMSGGRVPRVPASSTTTSASEGIFRRLVRRLRRGRGTADGAGVADETHQGPRPPLRKRLAQHFRRLRGFF
GRLTPRWLSGLGRRAQRWWRGRQRPLLDPSFHGLEAGDSFMRDLLKREEELIGYCREEALKEPAAMVEA
VMATVWPQNAETTVDSLLSQGERKLKLVEPLRVGDRSVVFLVRDVERLEDFALKVFTMGAENSRSELER
LHEATFAAARLLGESPEEARDRRRLLLPSDAVAVQSQPPFAQLSPGQSDYAVANYFFLMPAASVDLELLFRT
LDFVYVFRGEEGILARHLLTAQLIRLAANLQSKGLVHGRFTPENLFIMPDGRLMMGDVSTLRKVGTRGPA
SSVPVTYAPREFLNANTATFTHALNAWQLGLSIYRVWCLVLPFGLVTPGIKRTWKRPSLRVPGTDSLLFDSC
IPVPDFVQTLTRRFLNFDRRRRLLPLEAMETPEFLQLQNEISSSLSTGQPTAAPSVA.

>ROP5 ΔPD1(Δ1-115aa)

ETHQGPRPPLRKRLAQHFRRLRGFFGRLTPRWLSGLGRRAQRWWRGRQRPLLDPSFHGLEAGDSFMRDL
LKREEELIGYCREEALKEPAAMVEAVMATVWPQNAETTVDSLLSQGERKLKLVEPLRVGDRSVVFLVRDV
ERLEDFALKVFTMGAENSRSELERLHEATFAAARLLGESPEEARDRRRLLLPSDAVAVQSQPPFAQLSPGQS
DYAVANYFFLMPAASVDLELLFRTLDFVYVFRGEEGILARHLLTAQLIRLAANLQSKGLVHGRFTPENLFIM
PDGRLMMGDVSTLRKVGTRGPASSVPVTYAPREFLNANTATFTHALNAWQLGLSIYRVWCLVLPFGLVTP
GIKRTWKRPSLRVPGTDSLLFDSCIPVPDFVQTLTRRFLNFDRRRRLLPLEAMETPEFLQLQNEISSSLSTGQ
PTAAPSVA.

>ROP5 ΔPD2(Δ133-143aa)

MATKLARLATWLVLVGCLLWRAGAVQLSPPNSRTNDLASGTPHVARGDTEAQSGTGDDSDFPQGVVEEV
ADMSGGRVPRVPASSTTTSASEGIFRRLVRRLRRGRGTADGAGVADETHQGPRPPLRKRLAQHTPRWLSG
LGRRAQRWWRGRQRPLLDPSFHGLEAGDSFMRDLLKREEELIGYCREEALKEPAAMVEAVMATVWPQN
AETTVDSLLSQGERKLKLVEPLRVGDRSVVFLVRDVERLEDFALKVFTMGAENSRSELERLHEATFAAAR
LLGESPEEARDRRRLLLPSDAVAVQSQPPFAQLSPGQSDYAVANYFFLMPAASVDLELLFRTLDFVYVFRGE
EGILARHLLTAQLIRLAANLQSKGLVHGRFTPENLFIMPDGRLMMGDVSTLRKVGTRGPASSVPVTYAPRE
FLNANTATFTHALNAWQLGLSIYRVWCLVLPFGLVTPGIKRTWKRPSLRVPGTDSLLFDSCIPVPDFVQTLT
RRFLNFDRRRRLLPLEAMETPEFLQLQNEISSSLSTGQPTAAPSVA.

>ROP5 ΔPD3(Δ152-164aa)

MATKLARLATWLVLVGCLLWRAGAVQLSPPNSRTNDLASGTPHVARGDTEAQSGTGDDSDFPQGVVEEV
ADMSGGRVPRVPASSTTTSASEGIFRRLVRRLRRGRGTADGAGVADETHQGPRPPLRKRLAQHFRRLRGFF
GRLTPRWLSGLPLLDPSFHGLEAGDSFMRDLLKREEELIGYCREEALKEPAAMVEAVMATVWPQNAETTV
DSLLSQGERKLKLVEPLRVGDRSVVFLVRDVERLEDFALKVFTMGAENSRSELERLHEATFAAARLLGESP
EEARDRRRLLLPSDAVAVQSQPPFAQLSPGQSDYAVANYFFLMPAASVDLELLFRTLDFVYVFRGEEGILAR
HLLTAQLIRLAANLQSKGLVHGRFTPENLFIMPDGRLMMGDVSTLRKVGTRGPASSVPVTYAPREFLNAN
TATFTHALNAWQLGLSIYRVWCLVLPFGLVTPGIKRTWKRPSLRVPGTDSLLFDSCIPVPDFVQTLTRRFLN
FDRRRRLLPLEAMETPEFLQLQNEISSSLSTGQPTAAPSVA.

>ROP5 ΔPD4(Δ501-518aa)

MATKLARLATWLVLVGCLLWRAGAVQLSPPNSRTNDLASGTPHVARGDTEAQSGTGDDSDFPQGVVEEV
ADMSGGRVPRVPASSTTTSASEGIFRRLVRRLRRGRGTADGAGVADETHQGPRPPLRKRLAQHFRRLRGFF
GRLTPRWLSGLGRRAQRWWRGRQRPLLDPSFHGLEAGDSFMRDLLKREEELIGYCREEALKEPAAMVEA
VMATVWPQNAETTVDSLLSQGERKLKLVEPLRVGDRSVVFLVRDVERLEDFALKVFTMGAENSRSELER
LHEATFAAARLLGESPEEARDRRRLLLPSDAVAVQSQPPFAQLSPGQSDYAVANYFFLMPAASVDLELLFRT
LDFVYVFRGEEGILARHLLTAQLIRLAANLQSKGLVHGRFTPENLFIMPDGRLMMGDVSTLRKVGTRGPA
SSVPVTYAPREFLNANTATFTHALNAWQLGLSIYRVWCLVLPFGLVTPGIKRTWKRPSLRVPGTDSLLFDSC
IPVPDFVQEAMETPEFLQLQNEISSSLSTGQPTAAPSVA.

PD1 PD2 PD3 PD4

B

Confidently predicted domains, repeats, motifs and features:

Name	Start	End	E-value
low complexity	95	115	N/A
low complexity	133	143	N/A
low complexity	152	164	N/A
low complexity	501	518	N/A

Click on a row to highlight the feature in the diagram above. Click the feature name for more information.

Figure S3 | The sequences and information for the deletion mutants of ROP5 (A) The amino acid sequences of full-length and deletion mutants of ROP5(The putative domains were highlighted). (B) The information of the putative domains identified in ROP5. This information were analyzed with online tools (http://smart.embl-heidelberg.de/(accessed on 3 August 2021)).

Table S1 | primers used in this study

Primers	Sequences from 5′ to 3′	Used to
ROP5-F<br>ROP5-R	ATAGGATCCATGGCGACGAAGCTCGCTAGACTAG<br>ATACTCGAGTCAAGCGACTGAGGGCGCAGCAGT	Amplify sequence of ROP5
mcGAS-F<br>mcGAS-R	AATGGTACCATGGAAGATCCGCGTAGAAGGACG<br>CCACTCGAGTCAAAGCTTGTCAAAAATTGGAAAC	Amplify sequence of cGAS
mSTING-F<br>mSTING-R	CCAGAATTCTATGCCATACTCCAACCTGCATCCAG<br>TTACTCGAGTCAGATGAGGTCAGTGCGGAGTG	Amplify sequence of STING
mTBK1-F<br>mTBK1-R	ATCGGATCCATGCAGAGCACCTCCAACCAT<br>AATGGGCCCCTAAAGACAGTCCACATTGCG	Amplify sequence of TBK1
mIRF3-F<br>mIRF3-R	CCAGAATTCTATGGAAACCCCGAAACCGCG<br>CCACTCGAGTCAGATATTTCCAGTGGCCTG	Amplify sequence of IRF3
R5ΔPD1-F<br>R5ΔPD1-R	ATAGGATCCATGCGGAAGAGACTTGCTCAGCACTTC<br>ATACTCGAGTCAAGCGACTGAGGGCGCAGCAGT	Amplify truncated ROP5
R5ΔPD2-F<br>R5ΔPD2-R	GAAGAGACTTGCTCAGCACACGCCGAGGTGGCTCTCCGGT<br>ACCGGAGAGCCACCTCGGCGTGTGCTGAGCAAGTCTCTTC	Amplify truncated ROP5
R5ΔPD3-F<br>R5ΔPD3-R	AGGTGGCTCTCCGGTCTCCCGCTGCTGGACCCTTCGTTTCAT<br>TGAAACGAAGGGTCCAGCAGCGGGAGACCGGAGAGCCACCT	Amplify truncated ROP5
R5ΔPD4-F<br>R5ΔPD4-R	ATAGGATCCATGGCGACGAAGCTCGCTAGACTAG<br>ATACTCGAGCTGCACGAAGTCAGGCACAGGTAT	Amplify truncated ROP5
qIFN-β-F<br>qIFN-β-R	CAGCTCCAAGAAAGGACGAAC<br>GGCAGTGTAACTCTTCTGCAT	qPCR analyses
qISG56-F<br>qISG56-R	CTGAGATGTCACTTCACATGGAA<br>GTGCATCCCCAATGGGTTCT	qPCR analyses
qIL-6-F<br>qIL-6-R	TAGTCCTTCCTACCCCAATTTCC<br>TTGGTCCTTAGCCACTCCTTC	qPCR analyses
qCXCL-10-F<br>qCXCL-10-R	CCAAGTGCTGCCGTCATTTTC<br>GGCTCGCAGGGATGATTTCAA	qPCR analyses
qISG15-F<br>qISG15-R	GGTGTCCGTGACTAACTCCAT<br>TGGAAAGGGTAAGACCGTCCT	qPCR analyses
qActb-F<br>qActb-R	GGCTGTATTCCCCTCCATCG<br>CCAGTTGGTAACAATGCCATGT	qPCR analyses
qTuBa-F<br>qTuBa-R	AGCTCTTCTGCTTGGAGCAC<br>GTTAGCTGCATCCTCCTTGC	qPCR analyses
qTBP-F<br>qTBP-R	GCCTTCCACCTTATGCTCA<br>TGTGTGGGTTGCTGAGATGT	qPCR analyses
qGAPDH-F<br>qGAPDH-R	AGGTCGGTGTGAACGGATTTG<br>TGTAGACCATGTAGTTGAGGTCA	qPCR analyses
qTgB1-F<br>qTgB1-R	AACGGGCGAGTAGCACCTGAG<br>TGGGTCTACGTCGATGGCATGACAAC	qPCR analyses
gRNA-F<br>gRNA-R	GATCGGTCACCGACTCGAAGGTTTTAGAGCTAGAAATA<br>AACTTGACATCCCCATTTAC	Construct gRNA plasmid

pUC19-F<br>pUC19-R	GGCGTAATCATGGTCATAGC<br>ACTGGCCGTCGTTTTACAAC	Amplify linerized vector
DHFR-F<br>DHFR-R	CAACCCGCGCAGAAGACATC<br>GGACACGCTGAACTTGTGGC	Amplify UTR sequence
5′UTR-F<br>5′UTR-R	GTTGTAAAACGACGGCCAGTAATACACATCCTGCATGCCG<br>GATGTCTTCTGCGCGGGTTGCTGGCAGATGTTGTGAGGTC	Amplify homologous
3′UTR-F<br>3′UTR-R	GCCACAAGTTCAGCGTGTCCGAAAACGGCTCTGCCAACGG<br>GCTATGACCATGATTACGCCACTGTACACACGACTACCAG	Amplify homologous
PCR1-F<br>PCR1-R	GGTTTCTGAGTGGTGACAGA<br>ATTTGTGAGGACGACTCACG	Identify ROP5 knockout strain
PCR2-F<br>PCR2-R	AGGATGAATTCCTACCCAGC<br>TCTCTAAACGGTATGGTGCC	Identify ROP5 knockout strain
PCR3-F<br>PCR3-R	AGTTGCTGACGAAACCCATC<br>TTTTCAGGTGTGAAGCGTCC	Identify ROP5 knockout strain
